# Multidrug-Resistant *Escherichia coli* Causing Respiratory and Systemic Infection in a Guinea Pig (*Cavia porcellus*) in Romania: A Case Report

**DOI:** 10.3390/vetsci13040370

**Published:** 2026-04-11

**Authors:** Alexandru Gligor, Vlad Iorgoni, Paula Nistor, Sebastian Alexandru Popa, Ionela Popa, Ionica Iancu, Ileana Nichita, Kalman Imre, Emil Tîrziu, Viorel Herman

**Affiliations:** 1 Department of Infectious Diseases and Preventive Medicine, Faculty of Veterinary Medicine, University of Life Sciences “King Mihai I” from Timişoara, 300645 Timişoara, Romania; alexandru.gligor@usvt.ro (A.G.); paula.nistor@usvt.ro (P.N.); ionica.iancu@usvt.ro (I.I.); viorel.herman@fmvt.ro (V.H.); 2 Department of Animal Production and Veterinary Public Health, Faculty of Veterinary Medicine, University of Life Science “King Mihai I”, 300645 Timisoara, Romania; sebastian.popa@usvt.ro (S.A.P.); kalmanimre@usvt.ro (K.I.); 3 Department of Semiology, Faculty of Veterinary Medicine, University of Life Sciences “King Mihai I” from Timişoara, 300645 Timişoara, Romania; ionela.popa@usvt.ro; 4 Department of Microbiology, Faculty of Veterinary Medicine, University of Life Science “King Mihai I”, 300645 Timisoara, Romaniaemiltirziu@usvt.ro (E.T.); 5 Academy of Romanian Scientists (AOSR), Splaiul Independenței 3, Sector 5, 050094 Bucharest, Romania

**Keywords:** *Escherichia coli*, guinea pig, respiratory infection, multidrug resistance, septicemia, companion animals, One Health

## Abstract

Background/Objectives: Multidrug-resistant (MDR) *Escherichia coli* has increasingly been recognized as a pathogen capable of causing severe systemic infections in various animal species. However, reports describing respiratory and septicemic infections caused by MDR *E. coli* in guinea pigs remain scarce. The objective of this report was to describe the clinical, pathological, and microbiological findings associated with a fatal infection in a domestic guinea pig. Case Study: A 10-month-old female guinea pig (*Cavia porcellus*), kept as a companion animal in a household environment, presented with acute respiratory distress, lethargy, and anorexia, progressing rapidly to death within approximately 36 h of onset. Post-mortem examination revealed severe pulmonary congestion, diffuse inflammatory lesions in the trachea, and generalized vascular congestion in multiple organs. Bacteriological cultures obtained from lung and bone marrow samples yielded pure growth of *Escherichia coli*. Identification was confirmed using MALDI-TOF mass spectrometry. Antimicrobial susceptibility testing demonstrated resistance to several antibiotic classes, including β-lactams, fluoroquinolones, tetracyclines, sulfonamides, and phenicols, while susceptibility was retained only to aminoglycosides. Molecular analysis revealed the presence of virulence genes involved in adhesion and iron acquisition, supporting the pathogenic potential of the isolate. Conclusions: This report highlights the ability of MDR *E. coli* to cause severe respiratory and systemic infections in guinea pigs. The findings underline the importance of early diagnosis, appropriate antimicrobial stewardship, and improved husbandry conditions in preventing such infections. From a One Health perspective, the circulation of resistant strains in companion animals may represent a potential risk for both environmental and human health.

## Simple Summary

This report describes a fatal case of multidrug-resistant (MDR) *Escherichia coli* infection in a 10-month-old female domestic guinea pig in Romania. The animal presented with acute respiratory distress, lethargy, and anorexia, progressing to death within 36 h. Necropsy revealed severe pulmonary congestion, diffuse tracheal inflammation, and systemic vascular involvement. Cultures from lungs and bone marrow confirmed *E. coli*, resistant to multiple antibiotic classes (β-lactams, fluoroquinolones, tetracyclines, sulfonamides, phenicols) but susceptible to aminoglycosides. PCR identified virulence genes related to adhesion and iron acquisition, supporting pathogenic potential. The case illustrates the capacity of MDR *E. coli* to cause severe respiratory and systemic disease in guinea pigs. Environmental and host factors, such as stress or subtle immunosuppression, likely contributed to disease progression. The findings emphasize the importance of early diagnosis, targeted antimicrobial therapy, proper husbandry, and One Health awareness, given the potential public health implications of resistant bacterial strains in companion animals.

## 1. Introduction

Antimicrobial resistance (AMR) has emerged as a major global concern, affecting both human and animal medicine by significantly limiting the effectiveness of commonly used therapeutic agents. The continuous selection and dissemination of resistant bacterial strains, often mediated by mobile genetic elements, has contributed to the rapid spread of resistance determinants across different ecological niches. In this context, multidrug resistance (MDR), typically defined as resistance to at least three antimicrobial classes, represents a particularly challenging phenomenon due to its direct impact on treatment outcomes and infection control [1,2,3,4,5,6].

Among Gram-negative bacteria, *Escherichia coli* occupies a central position in both clinical and research settings. Although it is a normal inhabitant of the intestinal microbiota, certain strains have acquired specific virulence determinants that enable them to cause infections beyond the gastrointestinal tract. These extraintestinal pathogenic *E. coli* (ExPEC) strains are associated with a wide spectrum of conditions, including respiratory disease, septicemia, and urinary tract infections, affecting both animals and humans [1,7,8,9].

*Guinea pigs (Cavia porcellus)* are commonly maintained as companion animals as well as laboratory models. Despite their general adaptability, they remain susceptible to respiratory disorders, particularly under suboptimal environmental or management conditions. In this species, respiratory disease is most frequently linked to pathogens such as *Bordetella bronchiseptica* and *Streptococcus pneumoniae* [3,4,10,11,12,13,14]. However, opportunistic bacteria may also exploit compromised host defenses and contribute to disease development. Respiratory disease in guinea pigs may also be associated with pathogens such as *Pasteurella multocida, Pasteurella pneumotropica, Pneumocystis* spp., and adenoviruses, which should be considered in the differential diagnosis [12,13,15,16].

Several environmental and host-related factors may increase the susceptibility of guinea pigs to infectious diseases. Inadequate ventilation, excessive humidity, poor sanitation, and inappropriate bedding materials may negatively influence respiratory health. Furthermore, stress, nutritional imbalances, and insufficient veterinary care may impair immune competence, facilitating the transition of commensal or opportunistic microorganisms into pathogenic agents [3,12,13,14].

Although *E. coli* has been extensively investigated in a wide range of animal species, reports describing severe respiratory involvement associated with systemic dissemination in guinea pigs remain limited. The presence of MDR strains further complicates both diagnostic and therapeutic approaches, emphasizing the need for detailed case descriptions [3,4,15,16,17].

The present study aims to characterize a fatal case of respiratory and systemic infection caused by a multidrug-resistant *Escherichia coli* strain in a domestic guinea pig from Romania. Clinical findings, necropsy observations, microbiological investigations, and antimicrobial susceptibility testing were integrated to provide a comprehensive description of the case and to highlight its potential implications within a One Health framework.

To the best of our knowledge, this is the first report in Romania describing a multidrug-resistant *Escherichia coli* strain associated with both respiratory disease and septicemia in a guinea pig (*Cavia porcellus*), supported by the identification of specific resistance and virulence genes. This case provides additional insight into the pathogenic potential and epidemiological relevance of extraintestinal E. coli in less commonly studied companion species.

## 2. Case Study

This report describes a 10-month-old female guinea pig (*Cavia porcellus*) that was submitted for post-mortem examination to the Faculty of Veterinary Medicine in Timișoara, Romania. The animal was housed as a companion pet in a domestic environment along with two additional guinea pigs. According to the owner, the animals were kept indoors in a cage containing wood-chip bedding and were fed a diet primarily composed of commercial pellets, supplemented with fresh vegetables.

Although no major husbandry deficiencies were reported by the owner, several environmental and management-related factors that may predispose guinea pigs to opportunistic infections cannot be completely excluded. In particular, indoor housing conditions, including cage microclimate, ventilation efficiency, humidity levels, and potential accumulation of organic matter within bedding material, may have contributed to respiratory irritation and microbial proliferation. In addition, individual susceptibility, stress-related factors, or subtle immunosuppression could have facilitated the transition of *Escherichia coli* from a commensal organism to an opportunistic pathogen in this specific animal, while the other co-housed guinea pigs remained clinically unaffected.

Clinical signs were reported to have developed rapidly within approximately 24–36 h prior to death. Initially, the affected animal exhibited reduced activity and decreased food intake, followed by the onset of mild respiratory difficulty. The condition progressed quickly, with the guinea pig developing marked dyspnea, pronounced lethargy, and mucopurulent nasal discharge. No veterinary intervention was sought during this period, and the animal was found dead the following night.

A full necropsy was conducted shortly after presentation. Examination of the thoracic cavity revealed severe pulmonary alterations. The lungs were diffusely congested and edematous, displaying multiple dark-red to violaceous areas distributed across the lobes. The pulmonary tissue appeared moist and exhibited increased consistency on palpation, findings consistent with acute inflammatory consolidation.

**Figure 1:**
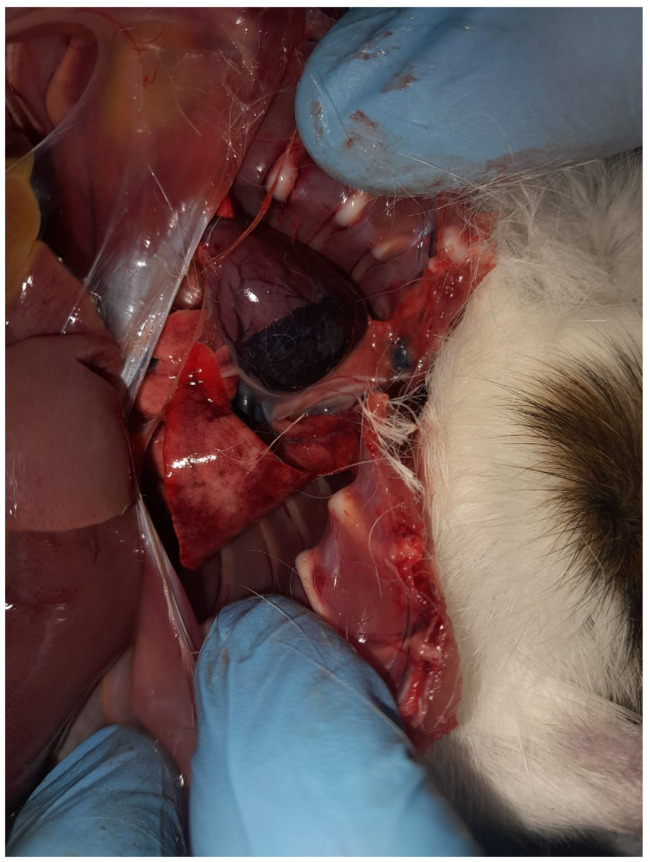
Acute congestive-hemorrhagic pneumonia, characterized by severe pulmonary congestion with multifocal hemorrhagic foci and edematous lung parenchyma.

Numerous hemorrhagic foci were observed beneath the pleural surface, predominantly affecting the cranial and middle lung lobes. In addition, areas of diffuse discoloration and parenchymal darkening were evident, suggesting extensive vascular involvement. The lesions showed a multifocal and uneven distribution pattern, indicative of an acute inflammatory process affecting the lungs. Overall, these findings were consistent with acute congestive-hemorrhagic pneumonia, with suspected systemic dissemination (Figure 1).

The tracheal mucosa was moderately hyperemic and contained a small amount of viscous mucoid exudate. Cardiac examination revealed a mild accumulation of serous fluid within the pericardial sac, without evident structural abnormalities. The abdominal organs, including the liver and spleen, exhibited moderate vascular congestion, supporting the presence of systemic circulatory disturbance.

Samples from the lungs and femoral bone marrow were collected aseptically for microbiological examination. The specimens were cultured on Columbia agar with 5% sheep blood and MacConkey agar and incubated at 37 °C under aerobic conditions. Primary evaluation was performed after 24 h, and cultures were further monitored up to 48 h. Samples were also cultured under microaerophilic conditions; however, no additional bacterial growth was observed.

Lactose-fermenting colonies consistent with *Escherichia coli* were observed on MacConkey agar, and subsequent subculture on eosin methylene blue agar revealed colonies with a characteristic metallic green sheen.

Bacterial identification was performed using MALDI-TOF MS with a score of 2.31. The obtained spectral profile was consistent with *Escherichia coli*; however, given the known limitation of MALDI-TOF MS in differentiating *Escherichia coli* from *Shigella* spp., the identification was supported by colony morphology, growth characteristics on selective media, and additional biochemical testing.

Antimicrobial susceptibility testing was carried out using the automated VITEK 2 system, in accordance with standardized laboratory procedures. The isolate demonstrated a multidrug-resistant profile, exhibiting resistance to multiple antimicrobial classes. Specifically, resistance was detected against β-lactams (ampicillin, amoxicillin-clavulanic acid, cefotaxime), fluoroquinolones (enrofloxacin, marbofloxacin), tetracyclines (doxycycline), phenicols (florfenicol), and sulfonamides (trimethoprim-sulfamethoxazole). Susceptibility was observed only for aminoglycosides, including gentamicin and amikacin (Table 1). The interpretation of antimicrobial susceptibility results was performed in accordance with the European Committee on Antimicrobial Susceptibility Testing (EUCAST) guidelines, using standardized breakpoints where applicable.

**Table 1:** Antimicrobial susceptibility profile of the *Escherichia coli* isolate, where R = Resistant; S = Susceptible.

Antimicrobial Class	Antimicrobial Agent	Result
β-lactams	Ampicillin	Resistant
β-lactams	Amoxicillin–clavulanic acid	Resistant
β-lactams	Cefotaxime	Resistant
Fluoroquinolones	Enrofloxacin	Resistant
Fluoroquinolones	Marbofloxacin	Resistant
Tetracyclines	Doxycycline	Resistant
Phenicols	Florfenicol	Resistant
Sulfonamides	Trimethoprim–sulfamethoxazole	Resistant
Aminoglycosides	Gentamicin	Susceptible
Aminoglycosides	Amikacin	Susceptible

Molecular characterization performed by PCR revealed the presence of several virulence-associated genes, including *fimH*, *papC*, *iutA*, and *ompA*, which are implicated in bacterial adhesion, immune evasion, and iron acquisition. Additionally, resistance genes such as *blaCTX-M*, *blaTEM*, *tetA*, and *sul1* were identified. All analyses were performed in a specialized microbiology laboratory. Molecular analysis was performed using PCR assays targeting selected virulence and antimicrobial resistance genes, as previously described [2,9,11,18,19].

The isolation of *Escherichia coli* from both pulmonary tissue and bone marrow confirmed the presence of systemic infection, consistent with septicemia. No additional bacterial or fungal pathogens were detected in the analyzed samples.

## 3. Discussion

*Escherichia coli* is widely recognized as a commensal component of the intestinal microbiota; however, specific strains possess genetic attributes that enable them to act as opportunistic pathogens in extraintestinal environments. These strains, collectively referred to as extraintestinal pathogenic *E. coli* (ExPEC), are capable of inducing a broad range of clinical conditions, including respiratory infections, septicemia, and systemic disease across multiple host species. Despite the extensive documentation of ExPEC in veterinary and human medicine, reports involving guinea pigs remain comparatively scarce [1,7,8,9].

The opportunistic nature of *Escherichia coli* infections should also be interpreted in the context of environmental and host-related factors. Although the animals were maintained under apparently standard domestic conditions, subtle deficiencies in microenvironmental parameters, such as inadequate ventilation, increased humidity, or suboptimal hygiene of bedding material, may have created favorable conditions for bacterial proliferation and respiratory tract colonization. Such factors are well recognized in guinea pigs, where respiratory health is highly sensitive to environmental quality [13,15,16].

Furthermore, the fact that only one individual was affected suggests a potential role of host-specific susceptibility, including transient immunosuppression, stress, or individual variation in microbiota composition. These elements may have facilitated the progression from colonization to systemic infection in this case.

In the present case, the isolation of *E. coli* from both pulmonary tissue and bone marrow provides strong evidence of systemic bacterial dissemination. All recovered isolates exhibited identical phenotypic antimicrobial susceptibility patterns, and genotypic analysis confirmed a uniform resistance profile across all samples. The detection of the pathogen in bone marrow is particularly indicative of hematogenous spread and supports the diagnosis of septicemia. Similar dissemination patterns have been described in other animal species, where ExPEC strains demonstrate the capacity to invade multiple organ systems and induce severe clinical outcomes [8,17,18,19,20,21,22].

The multidrug-resistant phenotype identified in this isolate highlights the ongoing expansion of antimicrobial resistance among bacterial pathogens affecting companion animals. Resistance to multiple antimicrobial classes, including β-lactams, fluoroquinolones, tetracyclines, and sulfonamides, significantly limits therapeutic options and complicates clinical management. From an epidemiological perspective, the presence of MDR bacteria in companion animals is of particular concern due to the close and frequent interactions between animals and humans, which may facilitate the exchange of resistant microorganisms.

In guinea pigs, the use of aminopenicillins is contraindicated due to their severe disruption of the intestinal microbiota, which may lead to fatal enterotoxemia. In this context, the observed resistance of the isolate to β-lactams does not have direct therapeutic implications, as these antimicrobials are generally avoided in this species. However, the susceptibility to aminoglycosides is clinically relevant, as these agents may represent a safer therapeutic option. Nevertheless, the overall multidrug-resistant profile of the isolate significantly limits available treatment choices and highlights the importance of targeted antimicrobial therapy [23,24,25].

The molecular detection of virulence-associated genes such as *fimH*, *papC*, *iutA*, and *ompA* further supports a profile consistent with an ExPEC-like strain, although full classification based on established criteria cannot be definitively confirmed. These genetic determinants are involved in key pathogenic mechanisms, including adhesion to host tissues, evasion of host immune responses, and acquisition of essential nutrients such as iron. Their combined presence likely contributed to the invasive capacity of the strain and to the rapid progression of disease observed in this case [22,26,27,28,29,30,31,32].

The identification of resistance genes, including *blaCTX-M*, *blaTEM*, *tetA*, and sul1, underscores the genetic basis underlying the observed antimicrobial resistance profile. These genes are frequently associated with mobile genetic elements, which facilitate horizontal gene transfer between bacterial populations. As a result, the potential dissemination of such determinants within the household environment cannot be excluded. From a One Health perspective, the detection of a multidrug-resistant ExPEC like strain in a companion guinea pig raises important considerations regarding the role of small mammals as potential reservoirs of antimicrobial resistance. Although direct transmission to humans was not investigated in this case, the close proximity between pets and their owners creates opportunities for bidirectional exchange of microorganisms. Consequently, infections involving MDR bacteria in companion animals should not be regarded as isolated events but rather as components of a broader ecological system linking animal, human, and environmental health [26,27,28,29,33].

Interestingly, the isolate remained susceptible to aminoglycosides despite exhibiting resistance to multiple other antimicrobial classes. This finding may be explained by the different mechanisms of action and resistance involved. While tetracyclines and phenicols act on the 30S and 50S ribosomal subunits through mechanisms often associated with efflux pumps or ribosomal protection, aminoglycosides induce irreversible inhibition of protein synthesis and are less affected by these resistance determinants. The absence of specific aminoglycoside-modifying enzymes may account for the preserved susceptibility observed in this case [1,34,35].

Similar susceptibility patterns have been reported in other animal species, where multidrug-resistant *Escherichia coli* isolates retain sensitivity to aminoglycosides despite broad resistance to other antimicrobial classes. This highlights the potential clinical relevance of this class in selected cases, although its use must be carefully considered due to species-specific toxicity and administration constraints [1,36,37,38].

From a One Health perspective, several hypotheses may explain the rapid evolution of the disease and the absence of clinical signs in the other co-housed animals. The route of infection likely involved either respiratory or oral exposure, possibly through contaminated bedding, feed, or water. The hyperacute progression suggests a combination of increased bacterial virulence and individual host susceptibility. The presence of multiple virulence-associated genes supports the invasive potential of the strain, while host-related factors such as stress or transient immunosuppression may have facilitated disease progression in this specific animal.

A concurrent or prior viral infection cannot be excluded, as such conditions may impair respiratory defenses and predispose to secondary bacterial infections. Similar patterns have been described in other animal species, where opportunistic *Escherichia coli* strains can cause severe systemic disease under favorable environmental or host-related conditions. These findings highlight the complex interplay between pathogen, host, and environment within the One Health framework.

This study is subject to certain limitations. The description of a single clinical case restricts the generalizability of the findings and does not allow for broader epidemiological conclusions. Additionally, the absence of whole-genome sequencing limits the depth of genetic characterization of the isolate, particularly with regard to phylogenetic classification and the identification of additional resistance or virulence determinants. Future investigations incorporating larger sample sizes and advanced molecular techniques would contribute to a more comprehensive understanding of ExPEC infections in guinea pigs and other companion species.

A limitation of the present report is the absence of histopathological examination, which could have provided a more detailed characterization of the pulmonary lesions and a better understanding of tissue-level pathological changes. Although the gross findings were strongly suggestive of acute inflammatory and hemorrhagic processes, microscopic evaluation would have allowed a more precise assessment of lesion severity and distribution, as well as confirmation of bacterial involvement at the tissue level.

An additional limitation is the lack of comprehensive characterization required for definitive classification of the isolate as extraintestinal pathogenic *Escherichia coli* (ExPEC), according to previously established criteria. Therefore, the strain should be interpreted as exhibiting an ExPEC-like profile rather than being conclusively classified within this pathotype.

Overall, the present findings emphasize the ability of multidrug-resistant *Escherichia coli* to cause severe respiratory and systemic disease in guinea pigs. Continuous monitoring of antimicrobial resistance patterns in companion animals, including less frequently studied species, remains essential for improving both clinical management and public health surveillance.

## 4. Conclusions

This case report demonstrates that multidrug-resistant *Escherichia coli* can cause severe respiratory disease and septicemia in guinea pigs. The rapid progression of the infection and the extensive antimicrobial resistance profile highlight the challenges associated with diagnosing and managing such infections.

Improved surveillance of antimicrobial resistance in companion animals, combined with responsible antibiotic use and enhanced biosecurity practices, is essential to mitigate the spread of resistant bacterial strains. From a One Health perspective, monitoring bacterial pathogens circulating in small mammals may contribute to a better understanding of antimicrobial resistance dynamics at the human–animal–environment interface.

## Data Availability

The original contributions presented in this study are included in the article. Further inquiries can be directed to the corresponding author.
